# A conversation with Mehdi Shishehbor, DO, MPH, PhD, President, Harrington Heart & Vascular Institute and Professor of Medicine, Cleveland University Hospitals Cleveland Medical Center

**DOI:** 10.1017/cts.2024.603

**Published:** 2024-10-24

**Authors:** Clinical Research Forum

**Affiliations:** Clinical Research Forum, Washington, DC, USA

## Top 10 Clinical Research Achievement Awards Q & A

This article is part of a series of interviews with recipients of Clinical Research Forum’s Top 10 Clinical Research Achievement Awards. This interview is with Mehdi Shishehbor, DO, MPH, PhD, President, Harrington Heart & Vascular Institute and Professor of Medicine, Cleveland University Hospitals Cleveland Medical Center. Dr Shishehbor’s area of expertise includes vascular medicine and minimally invasive, catheter-based procedures to reconstruct lower extremity arteries in order to treat critical limb ischemia and prevent amputation. He received a 2024 Top 10 Clinical Research Achievement Award for Transcatheter Arterialization of Deep Veins in Chronic Limb-Threatening Ischemia. *The interview has been edited for length and clarity.*

## How did you decide to pursue a career in clinical research?

People say, “Your mentors make you,” and that was certainly true for me. My first mentor was the renowned physician scientist Stanley Hazen, who took me under his wing while I was in my residency at the Cleveland Clinic. I had trained as a Doctor of Osteopathic Medicine and back then, DO programs typically didn’t offer much exposure to academic medicine. Dr. Hazen gave me the opportunity to do true translational research and helped make that happen in the middle of my residency – which wasn’t easy, considering how resident programs are structured. I just didn’t want to wait until the end of my residency to do research. What if I liked it? What if I didn’t? I felt if I was going to take time out to work in a lab, I wanted the experience to be as informative for my career as possible. Dr. Hazen supported me and to be honest, that year changed my life. It gave me not only the confidence but also the love and passion to pursue clinical research.

## What about that year was so inspirational?

My research in Dr. Hazen’s lab was focused on identifying novel markers of inflammation that correlated with clinical outcomes, and it turned out to be very productive, leading to the publication of three papers. So, I saw the whole process – from the basic science to publication and having my work recognized. But even more important, I saw that my research could have a direct impact on patients. That was exciting, and still is, because the markers we identified continue to be used clinically to prognosticate patients. The research I did that year also led me to two other mentors who have been extremely influential: Dr. Eric Topol, who was chairman of the Department of Cardiovascular Medicine at the Cleveland Clinic at the time, and Dr. Michael Lauer, who had many leadership roles at the Cleveland Clinic and is now Deputy Director for Extramural Research, NIH. They offered so much support and helped shape my career in cardiology and academic medicine.

## During this time, you were also pursuing other academic goals?

Yes, in that year, I realized I wanted to strengthen my clinical research skills so I got an MPH. Then, when I was a cardiology fellow, I was fortunate to be selected to receive a four-year NIH KL-2 grant, which supports early career investigators. That enabled me to pursue a PhD in epidemiology. These steps are all related, and that’s why that year of research was so important. It set the foundation. I eventually moved more toward outcomes research, and then when I became faculty, I became interested in clinical trials and innovation around devices and technologies, leading to interventional cardiology.

## Is this when you started to focus more on limb salvage research?

At first, my career was a bit all over the place, but then I started to focus on limb salvage. That’s because I saw how amputation could impact my patients. I was motivated to identify approaches that could identify and treat patients at risk of amputation. It was obvious that approximately 20–40% of patients with chronic limb-threatening ischemia were considered “no-option” patients and had no hope. These patients have no revascularization options. We can’t get through their calcified, totally occluded arteries with standard percutaneous or endovascular approaches. And until now, they used to get major amputations – that’s about 150,000 amputations every year in the United States. I have become extremely passionate about identifying solutions to help these patients. It is so important to do research in an area that you really care about, and for me, that is limb salvage, which is what the award-winning clinical trial is about.

## What did the results of that research demonstrate?

We found that transcatheter arterialization of the deep veins was safe and could be performed successfully in patients with chronic limb-threatening ischemia and no conventional surgical or endovascular revascularization treatment options. We used an investigational device which has since been approved by the FDA [US Food and Drug Administration]. It is actually the first device to get FDA approval for this condition and it is now available commercially.

## So this trial is already changing clinical practice?

Yes. There was such an unmet need. Until now, there were no medications or biologic therapies or devices that have been specifically approved for this condition. This device is the first one. It allows oxygenated blood to reach the distal foot by way of the venous system while addressing the limitations of surgical arterialization of deep veins.

## Where is this research headed next?

I’ve been talking to you about new technologies, but sometimes the next step is not about a new medical device or procedure. As I see it, the next step in this field should be about improvements in care delivery. That’s the area that has lagged significantly for these patients. Chronic limb-threatening ischemia is a complex challenge. A total of 80% of people with this condition have diabetes. In total, 40% have chronic kidney disease. Twenty percent are on dialysis. When a patient comes to me, they typically have had some tissue loss on their foot, and doing a procedure to care for that and save their limb is just the beginning of the long road ahead. They require significant podiatry and wound care. They need to control their other co-morbidities. They need risk factor modification. But the current health system does not support that; the care delivery model is not there for these patients. That can be so frustrating. I can spend five hours doing a very complicated operation and then afterward, things fall apart.

## What can be done to improve the care delivery model for these patients?

One of the things that we have done here is to hire a care coordinator. This person is dedicated to the care of these patients and helps ensure that the procedures we do actually lead to healing. It’s part of taking a team-based approach. Typically, medicine is very siloed, but we need to get beyond that and build teams that are willing to work together to deliver the highest level of care. With that in mind, four years ago, we established the Limb Salvage Advisory Council (LSAC).

## What is the role of the Limb Salvage Advisory Council (LSAC)?

The LSAC is an interdisciplinary group comprised of vascular surgeons, endovascular interventionalists, vascular medicine specialists, podiatrists, and wound care experts. In our organization, which includes 10 acute care facilities, no patient can undergo an amputation without a full evaluation by this team. In other words, if a physician decides that a patient is a candidate for amputation, there is a hard stop and the LSAC is activated. The care coordinator I just mentioned schedules a Zoom meeting and the members of the LSAC confer, from wherever we are in the world. We discuss the patient and make recommendations. In the first year of this program, the data showed that in our organization, 95% of the time when a patient was told that there were no options available, we were able to actually identify an option and do a procedure. Seventy-five percent of the time we were able to save the limb. It is an incredibly successful improvement to care delivery.

## How do you balance clinical practice, research, and other demands on your time?

I’m very lucky. I have two wonderful children and a wonderful wife, and they keep me grounded. I also love what I do. To save somebody’s leg is so rewarding and I get so much positive feedback from my patients. When I see a patient *walk* into my office – that gives me so much motivation to keep going. So, I have a great social life with my family and friends, and I have a great job that gives me the opportunity to impact patients and also advance the field through research. Additionally, we have an amazing team here at University Hospitals Harrington Heart & Vascular Institute. They are as passionate about limb salvage as I am, together we strive to leave no limbs behind. We are very blessed.

